# Effects of Er:YAG laser pre-treatment on dentin structure and bonding strength of primary teeth: an in vitro study

**DOI:** 10.1186/s12903-020-01315-z

**Published:** 2020-11-10

**Authors:** Jun hui Wang, Kuan Yang, Bai ze Zhang, Zhi fei Zhou, Zi rui Wang, Xin Ge, Lu lu Wang, Yu jiang Chen, Xiao jing Wang

**Affiliations:** 1 State Key Laboratory of Military Stomatology and National Clinical Research Center for Oral Disease and Shaanxi Clinical Research Center for Oral Diseases, Stomatology Department of Children, School of Stomatology, The Fourth Military Medical University, No. 145, Changle West Road, Xincheng District, Xi’an, Shaanxi China; 2 Department of Orthodontics, College of Stomatology, Xi’an Jiaotong University, No. 98, Xiwu Road, Xincheng District, Xi’an, Shaanxi China; 3 Department of Stomatology, General Hospital of Tibet Military Region, Chinese People’s Liberation Army, Lhasa, Tibet China

**Keywords:** Er:YAG laser, Primary teeth, Dentin tubules, Peritubular dentin, Shear bond strength, Dentin tablets

## Abstract

**Background:**

To investigate the effects of Er:YAG laser pre-treatment on the dentin structure and shear bond strength of primary teeth.

**Methods:**

Dentin specimens were prepared using freshly extracted intact primary molars and divided randomly into four groups based on the surface treatment applied. The control and etchant groups received no treatment and conventional acid etching treatment, respectively, while the energy and frequency groups received laser surface treatment with variable energy (50–300 mJ) and frequency (5–30 Hz) parameters. The morphology was observed using scanning electron microscopy. The surface-treated dentin slices were bonded to resin tablets, followed by thermocycle treatment. The shear strength was determined using a universal testing machine and de-bonded surfaces were observed using a stereomicroscope.

**Results:**

SEM observation showed that the surface morphology of the dentin slices changed after etching as well as after Er:YAG laser pre-treatment with different energy and frequency values. The dentin tubules opened within a specific energy (50–200 mJ) and frequency (5–20 Hz) range. Beyond this range, the intertubular dentin showed cracks and structural disintegration. Shear strength tests showed no significant changes after acid etching. The shear strength increased significantly (*P* < 0.05) after Er:YAG laser pre-treatment compared with that of the control group. The shear strength increased within the same energy (50–200 mJ) and frequency (5–20 Hz) range as the tubule opening, but not significantly (*P* > 0.05). The most common mode of interface failure was adhesive (interface) failure, followed by mixed and resin cohesive failure.

**Conclusions:**

Pre-treatment using Er:YAG laser opens the dentinal tubules without the formation of a smear layer and improves the bonding strength between the primary teeth dentin and the resin composites.

## Background

In line with the improvement of living standards as a result of recent advancements, the concept of "painless and minimally invasive dentistry" has been widely adopted for the treatment of dental patients. For instance, laser therapy is widely used in the field of stomatology [1, 2] due to its promising benefits such as low noise, no vibration, and less heat, in accordance with the concept of painless and minimally invasive approaches [3]. The erbium-doped yttrium aluminum garnet (Er:YAG) laser and neodymium-doped yttrium aluminum garnet (Nd:YAG) laser are the most commonly used lasers in the field of stomatology. The Nd:YAG laser operates at a wavelength of 1064 nm, which is appropriate for intermittent laser emission with minimal heat accumulation and the prevention of excessive temperature increase. These characteristics of the Nd:YAG laser result in superior efficacy and a lower possibility of damaging adjacent tissues [4].

The wavelength of the Er:YAG laser is 2940 nm and the intensity of the short pulse type, which is mainly used for the ablation of hard tissues (bone and teeth), can reach 1000 W or higher [5]. The Er:YAG laser causes no pain or discomfort unlike traditional air turbine hand-held devices, making it especially suitable for the treatment of primary teeth in pedodontic patients. These features may help young children overcome their fear and reduce the difficulty of treatment [6]. In addition, primary teeth have thinner enamel and dentin, dentin tubules with smaller diameters, and lower density and mineral content than permanent teeth. All of these factors contribute to the lower bonding strength of composite materials to primary teeth compared to that of permanent teeth [7]. Although the Er:YAG laser is widely used for the treatment of permanent teeth [8], it is not often selected for the treatment of primary teeth due to the relatively short oral retention period of primary teeth [8]. Therefore, further research to improve the bond strength of resin composites to primary teeth can be of great significance. In the present study, the effects of conventional treatment and Er:YAG laser pre-treatment on the dentin structure of primary teeth and the shear bond strength (SBS) to resin composite were investigated. This study also provides an experimental and theoretical basis for encouraging the application of Er:YAG laser pre-treatment during clinical treatment of primary teeth among pediatric patients.

## Methods

### Preparation of dentinal slices and study groups

This study was approved by the institutional research ethics committee at the Stomatological Hospital of the Fourth Military Medical University, China (Approval No. IRB-REV-2018044). The children’s parents/guardians were informed about the purpose of the study and the inclusion of extracted teeth in the research and provided informed consent.

The present in vitro study was conducted using freshly extracted retained primary molars (n = 80) collected during the period of September 2018 to December 2018. Teeth were free from dental caries, defects, or restorations. The extracted primary molars were immediately placed in 0.9% normal saline. The teeth were cleaned of debris, calculus, and pigment using a scraper and ultrasound scalers within 24 h, and stored in distilled water at 4 °C. The teeth were used within 1 month after extraction in accordance with the recommendations of the International Organization for Standardization [9]. The tooth crown was sectioned into 1.5 mm-thick slices using a low-speed cutting machine (SYJ-150, Shenyang Kejing Automation Equipment Manufacturing Co., Ltd., China) positioned perpendicular to the long axis of the teeth under running water as a coolant. Two middle dentin slices (a radius of 5 mm and a thickness of 1.5 mm) were selected from each tooth (160 in total from 80 teeth). All dentin slices were polished with 800 mesh waterproof abrasive paper, washed with distilled water for 5 min, dried, and used in the experiment immediately.

The dentin slices were randomly divided into different groups and subgroups according to the surface treatment applied. The dentin slices in the control group received no further treatment. For the etchant group, the dentin slices were etched for 20 s using 350 g/L phosphoric acid (Gluma, USA) followed by thorough washing with plenty of water for 20 s. The dentin slices in the energy group (variable energy; frequency of 10 Hz) were irradiated using an Er:YAG laser (Fotona, Slovenia) with an R14 handle. The laser was adjusted to the multi-streaming processor (MSP) mode and an optical fiber (diameter 0.6 mm) was applied vertical to the surface of the sample for about 1 mm. This was followed by irradiation using the mesh scanning mode for 10 s at a frequency of 10 Hz and a wavelength of 2940 nm with 60% water, 40% steam, and variable energy (50 mJ, 100 mJ, 150 mJ, 200 mJ, 250 mJ, and 300 mJ). The dentin slices in the frequency group (variable frequency; energy of 100 mJ) were treated with an energy of 100 mJ and variable frequency (5 Hz, 10 Hz, 15 Hz, 20 Hz, 25 Hz, and 30 Hz) (Table 1).

**Table 1:** Description of study groups and respective surface treatments

Group	Subgroup	N	Description
Control group	–	20	No further treatment of dentin slices
Etchant group	–	20	Dentin slices were etched with 350 g/L phosphoric acid (Gluma, USA) for 20 s and washed thoroughly with plenty of water for 20 s to avoid excessive drying and to maintain wettability
Energy groups (Er:YAG laser treatment; variable energy; frequency of 10 Hz)	50 mJ group	10	Dentin slices were irradiated using an Er:YAG laser (Fotona, Slovenia) with the R14 handle. The laser was adjusted to the multi-streaming processor (MSP) mode and the optical fiber (diameter 0.6 mm) was applied vertical to the surface of the sample for about 1 mm, followed by irradiation using the mesh scanning mode for 10 s at a frequency of 10 Hz and wavelength of 2940 nm, with 60% water, 40% steam, and variable energy (50 mJ, 100 mJ, 150 mJ, 200 mJ, 250 mJ, and 300 mJ)
	100 mJ group	10	
	150 mJ group	10	
	200 mJ group	10	
	250 mJ group	10	
	300 mJ group	10	
Frequency groups (Er:YAG laser treatment; variable frequency; energy of 100 mJ)	5 Hz group	10	Dentin slices were irradiated using the Er:YAG laser with the R14 handle. The laser was adjusted to MSP mode and the optical fiber (diameter 0.6 mm) was applied vertical to the surface of the sample for about 1 mm, followed by irradiation using the mesh scanning mode for 10 s at an energy of 100 mJ and wavelength of 2940 nm, with 60% water, 40% steam, and variable frequencies (5 Hz, 10 Hz, 15 Hz, 20 Hz, 25 Hz, and 30 Hz)
	10 Hz group	10	
	15 Hz group	10	
	20 Hz group	10	
	25 Hz group	10	
	30 Hz group	10	

Dental resin composite (Z350, 3 M, MN, USA) was filled into cavities, piled into blocks, and cured using a light curing lamp (3 M, USA). The cured resin composite block was cut into slices (4 mm × 4 mm × 2 mm) with a low-speed cutting machine (SYJ-150, Shenyang Kejing Automation Equipment Manufacturing Co., Ltd., China) and polished in a polishing machine (UNIPOL-830, MTI Corporation, USA) using 400- and 600-grit silicon carbide paper until the resin block surface was bright.

The prepared specimens were examined under a stereomicroscope at 10X (MZ1500 stereomicroscope, Nikon, Japan). Specimens without defects (n = 128) were surface-treated with 800 mesh waterproof abrasive paper, washed using distilled water for 5 min, cleaned ultrasonically, and dried. Surface-treated dentin specimens from all groups (Table 1) were bonded to the prepared resin slices using a general resin binder (3 M, MN, USA). For this process, each dentin slice was embedded in a self-setting resin block (height: 50 mm, diameter: 20 mm) with the bonding surface of the dentin facing upward, clean from resin and contamination. Afterward, a dentin bonding agent (3 M, USA) was applied to the dentin surface. The surface was dried after 30 s and the prepared resin slice was bonded. Excess adhesive was removed carefully and light-curing was performed from the top, left, and right sides of the specimens for 20 s. All bonded specimens were immersed in water for 24 h before transferring to artificial saliva (Pharmacy Department, Stomatology Hospital, the Fourth Military Medical University, Xi’an) in a 37 °C water bath (Shanghai Jinghong Experimental Equipment Co., Ltd, China). All specimens were subjected to automatic cold and hot thermos-cycling (ZLR Automatic Cold and Hot Bath Circulator, Morida Test Equipment Co., Ltd., Tianjin) 500 times (5 °C cold and 55 °C hot and cold water for 30 s respectively, intermittently for 15 s) prior to further evaluation of the bond strength.

### Scanning electron microscope (SEM) analysis

In order to observe the effects of various treatments on the dentin surface, two dentin specimens were randomly selected from each group and analyzed with SEM (s-4800 Hitachi, Japan). The treated specimen surfaces were sprayed with gold after the surface morphology was observed.

### Shear bond strength testing

**Fig. 1:**
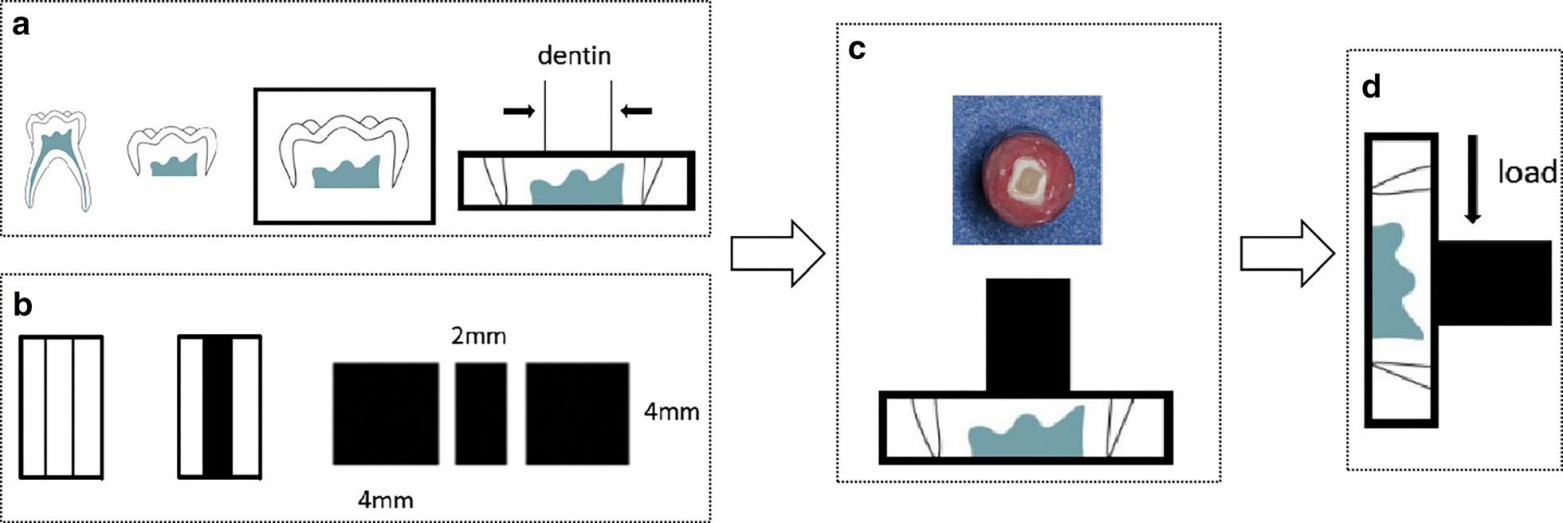
Preparation of dentin/resin slices and shear bond testing using a universal testing machine. **a** The crowns of the primary molars were cut at the level of cemento-enamel junction, sectioned in to 1.5 mm-thick slices, embedded into self-curing epoxy resin, ground and polished with 800 mesh waterproof abrasive paper. **b** Dental resin composite was filled into the cavity, piled up into blocks and light-cured, and cut into slices (4 mm × 4 mm × 2 mm) using a low-speed cutting machine and polished by 400–600-grit silicon carbide paper. **c** The resin slice was bounded to the dentin surface. **d** The shear bond strength was calculated by dividing the maximum load required to fail the bond by the bond area

The shear strength of the specimens were determined using a universal testing machine (AGS 500, Shimadzu, Japan) (Fig. 1). The testing was performed at room temperature (20 ± 2 °C), with the crosshead moving at a constant speed of 1 mm/min. The SBS was calculated as the maximum load value of the specimen at failure divided by the bond area. The bond failure mode was observed under a stereomicroscope (MZ1500 Stereomicroscope, Nikon, Japan).

### Statistical analysis

Data were processed and analyzed statistically using SPSS software (v22.0, IBM, USA). One-way analysis of variance (ANOVA) was used to analyze the count data (x ± s). The Kruskal Wallis test was used to compare the data and the rank sum test was used to measure the data. Statistical tests were set at a significance level of 5%.

## Results

### Morphology of dentin surface after different treatments

**Fig. 2:**
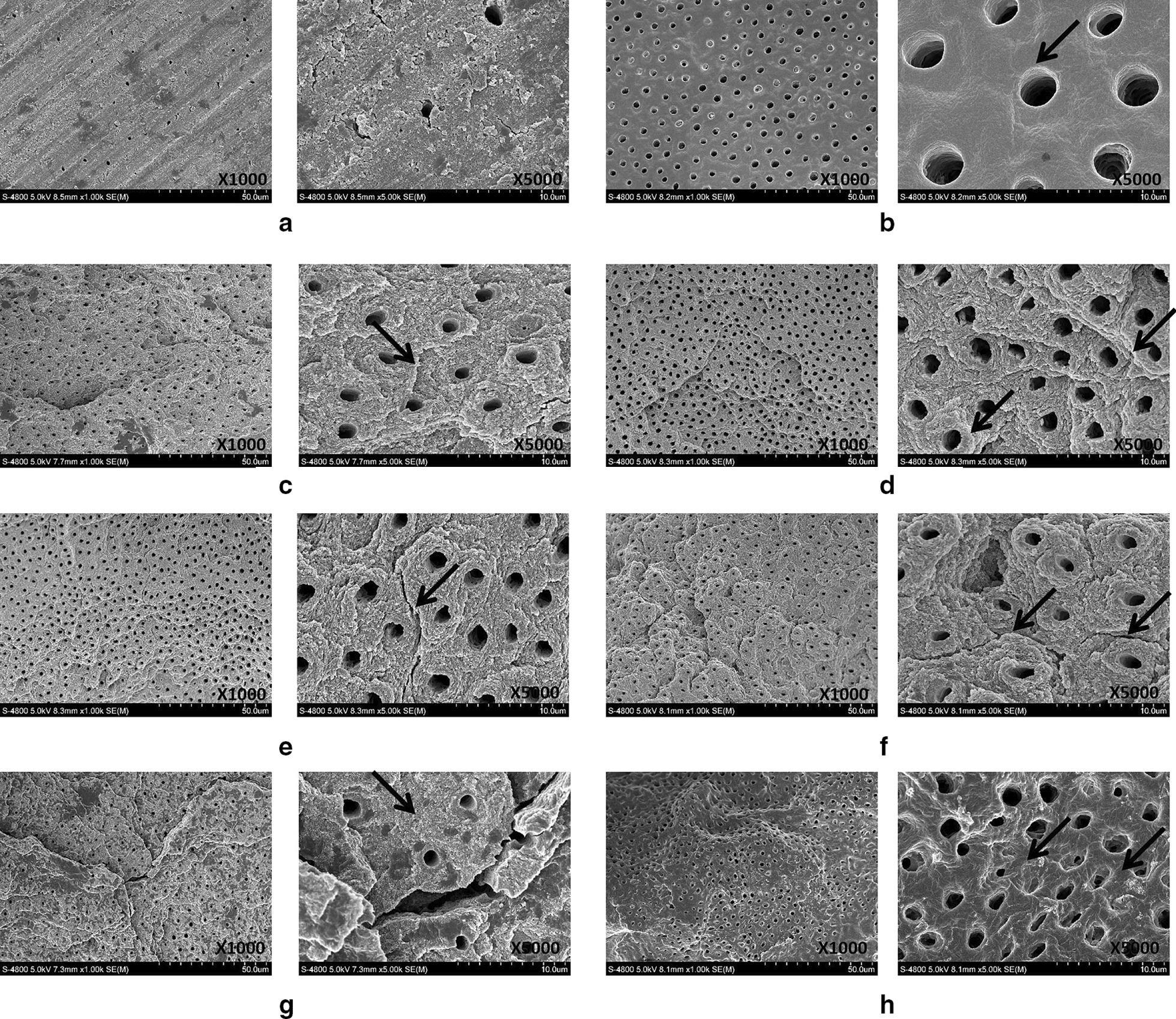
Representative SEM images showing morphology of dentin surface after different energy treatments. **a** Control group, **b** acid etching group, arrow indicates opened dentinal tubules, **c** 50 mJ laser group, arrow indicates protruding out of the peritubular dentin, **d** 100 mJ laser group, arrows indicate fish-scale and lamellar dentin, **e** 150 mJ laser group, arrow indicates periodontal dentin cracks, **f** 200 mJ laser group, arrows indicate peritubular dentin deep cracks, **g** 250 mJ laser group, arrow indicates disappearance of the fish-scale and lamellar morphology, **h** 300 mJ laser group, arrows indicate the collapsed tissues between the dentin tubes, coking, or carbonization

**Fig. 3:**
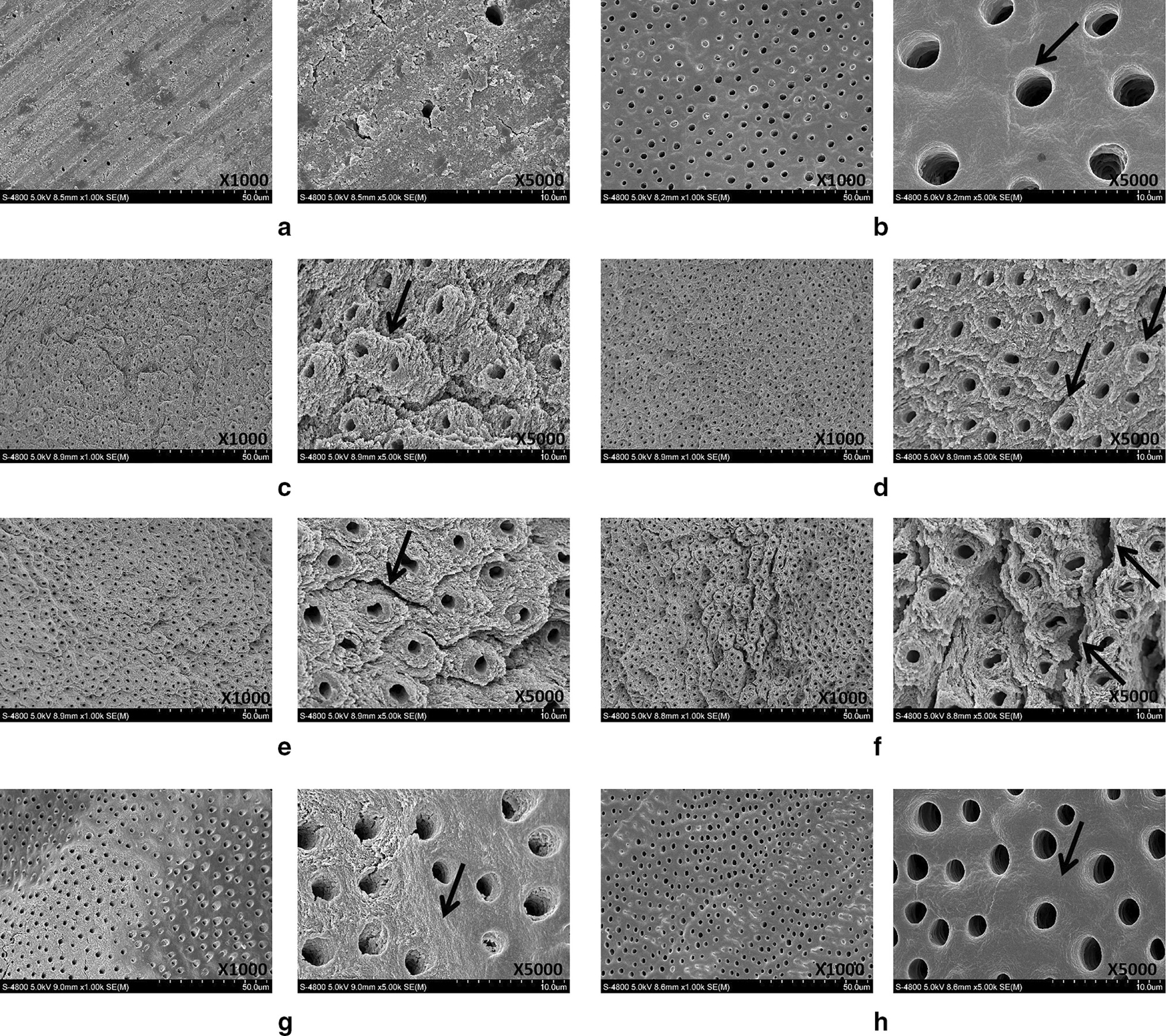
Representative SEM images showing morphology of dentin surface after treatment at different frequencies. **a** Control group **b** acid etching group, arrow indicates open dentin tubule, **c** 5 Hz laser group, arrow indicates protruding out of the peritubular dentin, **d** 10 Hz laser group, arrows indicate fish-scale and lamellar dentin, **e** 15 Hz laser group, arrow indicates periodontal dentin cracks, **f** 20 Hz laser group, arrows indicate periodontal dentin deep cracks, **g** 25 Hz laser group, arrow indicates the disappearance of the fish-scale and lamellar morphology and the boundaries between and around the tubes are unclear, **h** 30 Hz laser, arrow indicates the collapsed tissues between the dentin tubes, coking, or carbonization

The morphological changes in the surface dentin corresponding to various surface treatments were compared using SEM (Fig. 2, 3).

### Morphology of dentin surface following laser treatments at a specific frequency (10 Hz) and variable energy levels

In the control group, the dentin surface covered with thick smear layer, dentin tubule openings were blocked, and the boundary between the peritubular dentin and intertubular dentin was unclear (Fig. 2a). In the etching group, the dentin tubules were partially opened, and residual smear layers blocked some of the dentin tubules. The boundary between the peritubular dentin and intertubular dentin was not obvious. The tissues were completely collapsed between the dentin tubules and disorganization of the collagen fiber was observed, suggesting the presence of coking or carbonization (Fig. 2b). The 50 mJ group showed opening of the dentinal tubules and irregular dentin surface, with parts of the peritubular dentin protruding out of the surface, no smear layers blocked the dentin tubules (Fig. 2c). The dentin surface for the 100 mJ, 150 mJ, and 200 mJ groups showed open dentinal tubules, irregular dentin surface, no smear layers blocked the dentin tubules, and also exhibited a fish-scale and lamellar morphology. A few of these samples had a cuff-like appearance and the peritubular dentin protruded out of the surface (Fig. 2d–f). However, closer observation at 5000X revealed that the dentin tubules in the 150 mJ group were not completely round but circular and a few cracks in the peritubular dentin (Fig. 2e) extended between the dentinal tubules. In the 200 mJ group, it can be clearly observed that the deep crack penetrated through peritubular dentin (Fig. 2f). In the 250 mJ group, the fish-scale and lamellar appearance disappeared, resulting in a smoother dentin surface. The boundary was unclear between and around the tubules, the cracks in the peritubular dentin were enlarged, and the structures between the dentinal tubules were collapsed (Fig. 2g). The 300 mJ group showed morphological features very similar to those of the 250 mJ group; the tissues were collapsed completely between the dentin tubules and disorganization of the collagen fiber was observed, showing signs of coking or carbonization (Fig. 2h).

### Morphology of dentin surface following laser treatments at a specific energy level (100 mJ) and variable frequencies

The control and etching groups showed similar results as described earlier (Fig. 3a, b). In the 5 Hz, 10 Hz, and 15 Hz groups, the dentin surface had a fish-scale and lamellar appearance, irregular dentin surface, opened dentinal tubules, no smear layers blocked the dentin tubules, and protrusion of peritubular dentin from the surface (Fig. 3c–e). However, the 10 Hz group showed some cuff-like tubules (Fig. 3d) while the 15 Hz group showed a few cracks in the peritubular dentin (Fig. 3e).

In the 20 Hz groups, in addition to the fish-scale and lamellar appearance, the dentin tubules were open, not completely round but circular, and protruded out of the surface. Additionally, the cracks in the dentin tubule were deeper (Fig. 3f–h). In the 25 Hz group, the boundaries between and around the tubules were unclear, the cracks in the peritubular dentin were enlarged, a few cracks were visible at the orifice of the dentin tubules, and the dentinal structure had collapsed (Fig. 3g). In the 30 Hz group, the fish-scale and lamellar features disappeared with gross structural collapse in the dentin and peritubular dentin. A few areas even showed signs of coking or carbonization, and disorganization of the collagen fiber was observed, mimicking the morphology of the acid etching group (Fig. 3h).

### SBS after different treatments

#### Bonding strength at a specific frequency (10 Hz)

**Fig. 4:**
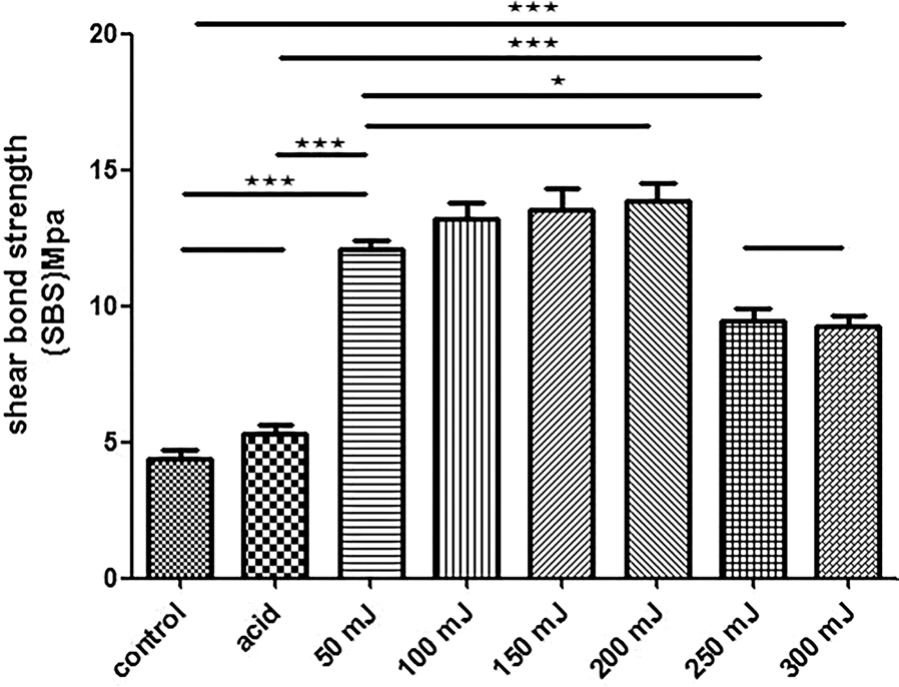
Comparison of shear bond strength of each group under different energy levels

After etching, the SBS was slightly increased; however, the difference was not statistically significant (*P* > 0.05). In contrast, Er:YAG laser pre-treatment increased the SBS significantly (*P* < 0.05). The SBS increased gradually but non-significantly when the energy of the laser increased from 50 to 200 mJ (Fig. 4). Increasing the Er:YAG laser energy to 250–300 mJ resulted in a gradual decrease in the SBS. The SBS values for the 50–200 mJ groups were significantly higher than those for the 250–300 mJ groups (*P* < 0.05) (Fig. 4).

#### Shear bond strength after different treatments at a specific energy (100 mJ)

**Fig. 5:**
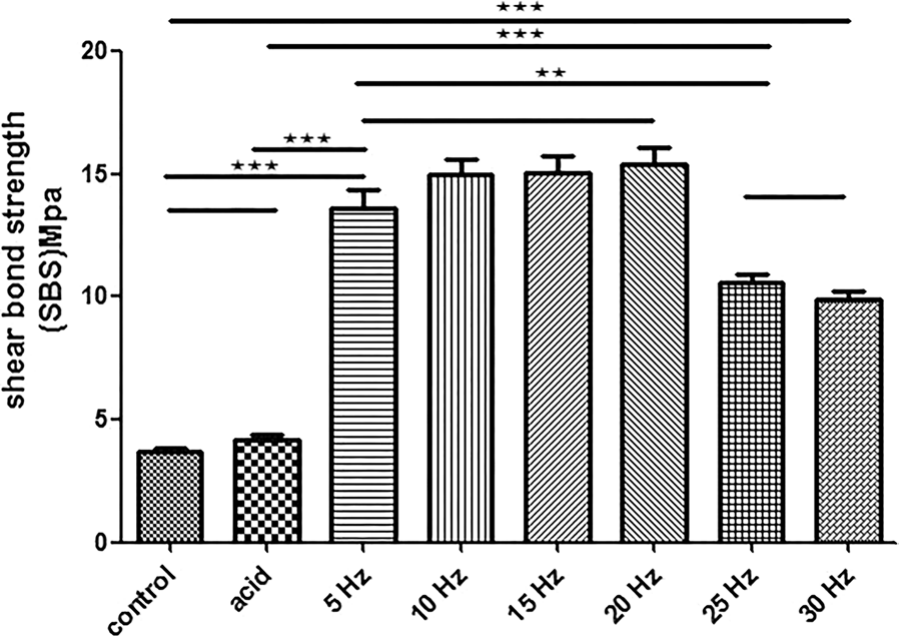
Comparison of shear bond strength of each group under different frequencies

The results showed that the SBS of the etching group remained unchanged compared to that of the Er:YAG laser groups, which showed significant increases (*P* < 0.05) in SBS (Fig. 5).

At a laser frequency of 5–20 Hz, the SBS increased gradually and the difference was not statistically significant (*P* > 0.05). However, increasing the frequency to 25–30 Hz reduced the SBS gradually. Overall, the SBS of the 5–20 Hz groups was significantly higher than that of the 25–30 Hz groups (*P* < 0.05) (Fig. 5).

#### Comparison of fracture modes

The fracture mode was mainly the interface fracture mode with both variable energy (76.56%) and variable frequency (78.13%), followed by the mixed fracture, resin cohesive fracture, and dentin cohesive fracture modes (Table 2).

**Table 2:** Comparison of fracture modes for various groups

Group	A, resin cohesive fracture	B, dentin cohesive fracture	C, interface fracture	D, mixed fracture	Total
*Various energy levels*					
Control	1	0	6	1	8
Acid	0	0	7	1	8
50 mJ	1	1	6	0	8
100 mJ	1	1	5	1	8
150 mJ	0	0	7	1	8
200 mJ	0	1	7	0	8
250 mJ	1	0	5	2	8
300 mJ	1	0	6	1	8
Total	5 (7.81%)	3 (4.69%)	49 (76.56%)	7 (10.94%)	64
*Various frequency levels*					
Control	1	0	6	1	8
Acid	1	0	7	0	8
5 Hz	0	0	8	0	8
10 Hz	0	0	8	0	8
15 Hz	1	1	5	1	8
20 Hz	1	0	6	1	8
25 Hz	1	1	5	1	8
30 Hz	1	1	5	1	8
Total	6 (9.38%)	3 (4.68%)	50 (78.13%)	5 (7.81%)	64

## Discussion

The effects of Er:YAG laser pre-treatment on the surface characteristics of dentin and the corresponding SBS to resin composite were investigated in the current study. For this purpose, dentin specimens prepared from extracted teeth were treated with conventional acid etching or Er:YAG laser under variable energy (50–300 mJ) and frequency (5–30 Hz) and the surface features and SBS were characterized. The experimental and theoretical results support the adoption of Er:YAG laser pretreatment during the clinical treatment of primary teeth among pediatric patients. Er:YAG laser pre-treatment remarkably changed the morphology of the dentin surface. The application of specific pre-treatment parameters such as energy level (50–200 mJ) and frequency (5–20 Hz) clearly opened the dentin tubules and induced the protrusion of the intertubular and peritubular dentin (Figs. 2, 3). Beyond this range, the dentin surface developed crack formations and structural disorientation. Similarly, the SBS increased significantly following Er:YAG laser pre-treatment, but decreased gradually beyond a certain range of energy and frequency values (Figs. 4, 5).

Air turbine hand-pieces are frequently used in clinical dentistry [10, 11]. Although these devices are simple to use and efficient for decay removal [12], the heat produced by mechanical friction may cause insult to pulpal tissues and pain. In addition, mechanical vibration and noise may aggravate stress and fear among young children, reducing their compliance with treatment and the quality of restorations [13]. Lasers are used for various restorative dentistry procedures and may potentially replace or minimize the use of air turbine hand-pieces [14, 15]. The advantages of lasers for clinical dental applications include low noise, no vibration, and less heat production [15]. In addition, there is almost no pain and discomfort to children during caries excavation and cavity preparation, making them useful tools for the treatment of carious primary teeth [15].

The Er:YAG laser is a solid-state laser that uses erbium, yttrium, aluminum, and garnet as the medium. The wavelength is 2940 nm, which is the absorption peak for water and hydroxyapatite [16]. The water and hydroxyapatite present in dental hard tissues absorb energy to form a "micro explosion", enabling the removal of water-containing tissues through energy absorption [17]. Effective tooth cutting can be selectively carried out to retain the maximum amount of healthy tooth tissues possible and to remove carious tissues. The laser used for treatment may have a substantial influence on the dentin surface morphology. The Er:YAG laser can enhance the bonding and interface between dentin and restorative materials [18, 19]. However, a few studies have reported that Er:YAG laser treatment reduces the bonding strength of dentin to resin composite [20, 21].

During cavity preparation, the dental pulp is sensitive to temperature changes; however, cavity temperature fluctuations of less than 5.5 °C degrees do not cause irreversible damage [22]. In addition, the ablation of dental hard tissues is affected by the intensity of the irradiation [23]. Ye et al. [23] reported that irradiation with a maximum power of 20 Hz and 6 W for 20 s increased the pulp cavity temperature by 3.05 ± 0.50 °C, which is well below the threshold temperature required for irreversible pulp damage. Therefore, a small dose and a short period of laser irradiation may prevent excessive removal and melting of healthy tooth tissues [23]. The power values selected in the current study were lower than 6 W, which ensured no excessive rise in the temperature during the treatment process and can be considered safe for pulp tissues. Referring to the parameter table for clinical applications of Fotona dental lasers and previous studies [8, 18, 23], laser frequency values of 5–30 Hz and energy values of 50–300 mJ were selected in the current study for the surface modification of dentin.

The SEM images showed that the use of the Er:YAG laser for dentin preparation results in a surface with a significantly different morphology compared to that achieved by conventional air turbine hand-held devices. The Er:YAG laser created an microretentive and irregular morphological pattern of the surface with open dentinal tubules and no smear layer. Within a specific energy (50–200 mJ) and frequency (5–20 Hz) range, the dentin tubules showed enhanced opening and protrusion of the intertubular surface dentin, which may promote the penetration of adhesive resin tags into the dentinal tubules and improve adhesion [24]. Further increases to the energy (250–300 mJ) and frequency (25–30 Hz) led to the formation of surface cracks and disintegration of the structural dentin. These findings are in agreement with previous studies [25, 26], which demonstrated that the Er:YAG laser at a certain energy and frequency range promotes the opening of dentinal tubules by effective removal of the smear layer [27]. Beyond this optimal range of energy and frequency, laser pre-treatment produced excessive energy and heat to the tissues. Initially, the Er:YAG laser vaporizes water and other hydrated organic components until internal pressure causes the destructive explosion of the inorganic component before the melting point is reached. Excessive energy and heat to the tissues finally leads to crack formation and dentin disintegration.

Corresponding to the surface changes, bond strength testing showed significantly increased SBS for the dentin slices bonded to resin composite following Er:YAG laser treatment. As the Er:YAG laser treatment was non-contact, no smear layer was formed on the dentin tubules, peritubular dentin, or intercellular dentin [25]. The Er:YAG laser treatment improved the dentin surface roughness and surface area by opening the dentinal tubules and facilitated the penetration of adhesive into the dentin tubules, such changes without a smear layer are believed to provide suitable surfaces for strong bonding with composite resin materials resulting in improved bonding [24]. In contrast, drilling dentin with an air turbine hand-piece leads to the formation of a smear layer on the surface of the dentin, blocking the dentinal tubules as well as interfering with the penetration of the resin adhesive and bonding with restorative materials. Therefore, to achieve better adhesion with tooth tissues, it is vital to reduce the formation of a smear layer and to remove the formed smear layer from the tooth surface. These findings are consistent with previous studies. Yazici et al. [19] reported that the application of the Er:YAG laser using a self-etching bonding system can increase the dentin SBS. In contrast, Ferreira et al. [28] indicated that dentin surfaces treated with the Er:YAG laser exhibited SBS values below that obtained with the traditional phosphoric acid etching treatment. This result may be related to the existence of a wide range of microcracks under the dentin surface after laser treatment. Acid etching is a technique-sensitive process and a minor variation in the etching time may change the surface and structural properties of the dentin [29]. Dunn et al. [30] suggested that the bonding strength of enamel following laser irradiation was significantly lower than that obtained with acid etching alone, possibly due to the high laser energy density (140 mJ/30 Hz). These variations in the bonding test results reported by different scholars suggest a lack of consensus, which is related to the use of variable parameter settings for laser treatment, laser brand selection, and type of adhesive [21, 31, 32].

In the present study, the SBS increased gradually within a specific range of Er:YAG laser energy (50–200 mJ). However, increasing the energy further gradually reduced the SBS. This outcome is associated with the disintegration and structural changes in dentin tubules corresponding to the excessive energy. Beyond this optimal range of energy and frequency, laser pre-treatment leads to crack formation and dentin disintegration. The presence of this fused layer without interfibrillar spaces was thought to restrict the resin diffusion into the subsurface intertubular dentin and thereby resulting in lower SBS. Ceballo et al. [33] and Koliniotou-Koumpia et al. [20] reported denaturing of the tooth surface collagen fiber following laser irradiation. The denaturing of collagen fibers reduces surface porosity, which is not conducive to the penetration of adhesive [34].

The formation of a fracture can be influenced by the bonding area. A larger bonding area may lead to the formation of more stress concentration points in the sample matrix, which facilitates internal fracture [35]. On the other hand, a smaller bonding area results in more uniform stress distribution, reducing the likelihood of cohesive fracture [36], which can reflect the true bond strength of the material. In the current study, 77.34% of the observed specimens had interface fractures (adhesive failures), followed by mixed fractures, which reflected the actual fracture modes for the dentin and resin. The data suggested that Er:YAG laser treatment (for all groups) made no significant difference in the failure mode compared to acid etching.

There are a few limitations worth noting. This in vitro study was carried out using healthy extracted teeth; therefore, the findings may be different for the oral environment during actual clinical applications. Our findings are based on healthy dentin, however, the effect of the Er:YAG laser on carious dentin may differ from that of normal dentin. Further experiments are required with different conditions and parameters.

## Conclusions

The treatment using Er:YAG laser predominately opened the dentinal tubules without the formation of a smear layer. Er:YAG laser pre-treatment with a specific range of energy and frequency improved the bonding strength between the dentin and the resin in primary teeth. The Er:YAG laser pre-treatment using a combination of 100 mJ and 10 Hz opened the dentinal tubules, resulting in a fish-scale and lamellar morphology and protrusion of the peritubular dentin from the surface. In addition, the SBS was relatively greater due to the removal of the smear layer and the alteration of dentin surface morphological features.

## Data Availability

The datasets used and/or analyzed during the current study are available from the corresponding author on reasonable request.

## Abbreviation

Er:YAG laser: Erbium: yttrium-aluminum-garnet laser
